# CX_3_CR1-CX_3_CL1-dependent cell-to-cell Japanese encephalitis virus transmission by human microglial cells

**DOI:** 10.1038/s41598-019-41302-1

**Published:** 2019-03-18

**Authors:** Nils Lannes, Obdullio Garcia-Nicolàs, Thomas Démoulins, Artur Summerfield, Luis Filgueira

**Affiliations:** 1 Unit of Anatomy, Department of Medicine, University of Fribourg, Route Albert-Gockel 1, Fribourg, Switzerland; 2 Institute of Virology and Immunology, Sensemattstrasse 293, Mittelhäusern, Switzerland; 3 Department of Infectious Diseases and Pathobiology, Vetsuisse Faculty, University of Bern, Langassstrasse 122, Bern, Switzerland

## Abstract

The neurotropic Japanese encephalitis virus (JEV) is responsible for Japanese encephalitis, an uncontrolled inflammatory disease of the central nervous system. Microglia cells are the unique innate immune cell type populating the brain that cross-communicate with neurons via the CX_3_CR1-CX_3_CL1 axis. However, microglia may serve as a viral reservoir for JEV. Human microglia are able to transmit JEV infectivity to neighbouring cells in a cell-to-cell contact-dependent manner. Using JEV-treated human blood monocyte-derived microglia, the present study investigates molecular mechanisms behind cell-to-cell virus transmission by human microglia. For that purpose, JEV-associated microglia were co-cultured with JEV susceptible baby hamster kidney cells under various conditions. Here, we show that microglia hosting JEV for up to 10 days were able to transmit the virus to susceptible cells. Interestingly, neutralizing anti-JEV antibodies did not completely abrogate cell-to-cell virus transmission. Hence, intracellular viral RNA could be a contributing source of infectious virus material upon intercellular interactions. Importantly, the CX_3_CL1-CX_3_CR1 axis was a key regulator of cell-to-cell virus transmission from JEV-hosting human microglia. Our findings suggest that human microglia may be a source of infection for neuronal populations and sustain JEV brain pathogenesis in long-term infection. Moreover, the present work emphasizes on the critical role of the CX_3_CR1-CX_3_CL1 axis in JEV pathogenesis mediating transmission of infectious genomic JEV RNA.

## Introduction

Japanese encephalitis (JE) is an uncontrolled inflammatory disease of the central nervous system (CNS) resulting from the infection by the neurotropic flavivirus, JE virus (JEV). JEV consists of a single stranded positive sense RNA (ssRNA+) encoding for 3 structural proteins (capsid protein (C), precursor to membrane protein (prM) and envelop protein (E)) and 7 non-structural proteins (NS1, NS2A, NS2B, NS3, NS4A, NS4B and NS5)^1^. Phylogenetic studies on prM suggest the presence of 5 genotypes for JEV^1^. JEV is transmitted by mosquito vectors in a zoonotic cycle including pig as amplifiers and water bird as reservoir hosts^2^. Humans are accidental dead-end hosts because of low viremia that does not allow further virus transmission^1^. In regions at risks, JE has an annual incidence of 70,000 symptomatic cases with 25–30% of mortality rate and 50% of survivors having life-treating neurological problems^3,4^. JEV is endemic in northern regions and epidemic in southern regions of the Asia-Pacific^5^. However, the detection of JEV in Europe^6,7^ and Africa^8^, the presence of competent vectors for JEV in Germany^9^ as well as the ability of JEV to persist and transmit between pigs in the absence of mosquitos^10^ are increasing risks for virus spread and persistence in regions with more moderate climate. Therefore, JE may become a worldwide health concern despite the establishment of efficient vaccines and vaccination programs^3^.

By a still unknown mechanism, JEV enters into the brain and targets neuronal cells with a specific tropism for developing neurons^11^. In particular, areas of neuronal turn-over, including the thalamus, the brainstem and the hippocampus, are the main brain regions of JEV–infected neurons found in brain autopsy studies of fatal JE patients^12^. In the CNS, microglial cells are a unique resident immune cell population able to migrate, phagocyte and present antigen upon insults^13,14^. Microglia develop during early development of the foetus, but can also derive from blood monocytes after birth under specific conditions^15^. In the JEV context, human microglia do not release infectious virus particles, but sustain viral RNA during a long period after virus exposure. However, microglia-associated virus remains infectious to susceptible cells under cell-to-cell contact conditions, allowing virus recovery^16^. Actually, microglia are proposed to play a possible role in long-lasting infection^17^.

Chemokines have potent chemotactic activities leading to the attraction or repulsion of specific cell types in various body compartments. In the CNS, the CX_3_CR1-CX_3_CL1 axis mediates the cross-communication between CX_3_CR1-expressing microglia and CX_3_CL1-expressing neurons^18^. In the CNS, CX_3_CR1-CX_3_CL1 maintains homeostasis and regulates inflammatory responses in compromised brain tissues^19^. Nevertheless, CX_3_CR1-CX_3_CL1 is protective in herpes simplex virus infection^20^ whereas it is detrimental in Theiler’s encephalomyelitis virus infection^21^. Microglia upregulates CX_3_CR1 expression in response to JEV exposure^16^, but the role of the CX_3_CR1-CX_3_CL1 axis remains unknown.

The present study aims to understand and dissect the mechanisms behind virus transmission and recovery from JEV-associated human microglia. In order to achieve this work, human monocyte-derived microglia were exposed to Nakayama JEV strain *in vitro* until supernatants were free of infectious virus. Virus recovery was subsequently achieved by adding susceptible target baby hamster kidney 21 (BHK-21) cells to JEV-associated microglia. Our results demonstrate that virus recovery from the target cells occurred upon cell contact-mediated virus transmission from JEV-associated microglia up to 10 days after virus exposure. Cell-to-cell virus transmission was not affected by the presence of neutralizing anti-JEV antibodies and *de novo* virus particles production by target cells could overcome neutralizing activities. Interestingly, viral RNA may be a contributing source of infectious virus material for cell-to-cell virus transmission. The latter virus transmission was dependent on CX_3_CR1-CX_3_CL1 interactions. Overall, the present study defines a novel function of human microglia as source of JEV virus infection via cell-to-cell virus transmission, independently of virus binding to its receptor on target cells.

## Results

### JEV recovery from JEV-pre-treated human microglia occurs at late periods after virus exposure

**Figure 1:**
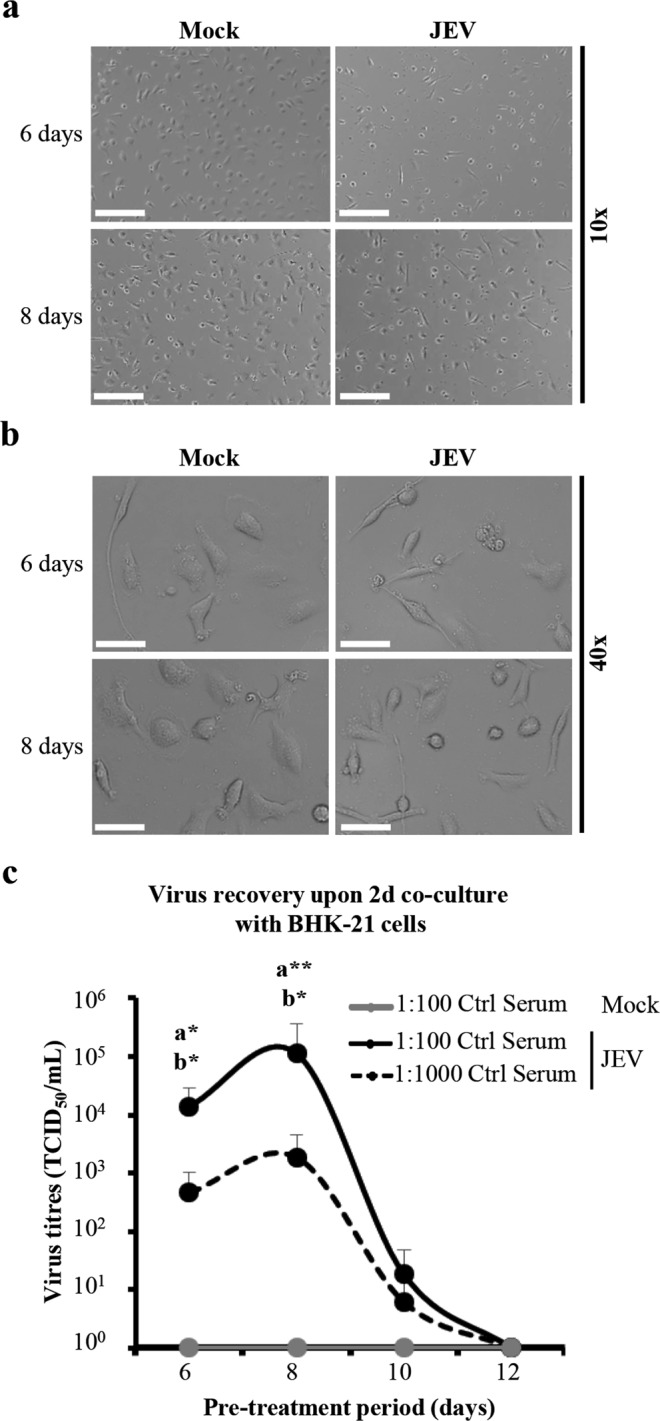
JEV recovery from JEV-pre-treated human microglia after late periods of exposure. (**a**,**b**) Human microglia were treated with Mock and JEV (Nakayama isolate used at a multiplicity of infection (MOI) of 10 TCID_50_/cell) at 37 °C for indicated time-periods. Representative micrographs showing cell monolayer and morphology at magnification of (**a**) 10x and (**b**) 40×. Scale bars are of (**a**) 200 μm and (**b**) 50 μm. In (**c**), human microglia were pre-treated with Mock and JEV (Nakayama isolate used at a MOI of 10 TCID_50_/cell) at 37 °C for indicated time-periods. The latter cell were intensively washed with cold PBS and subsequently co-cultured with BHK-21 cells for 2 additional days in the presence of indicated concentrations of control porcine serum (Ctrl serum). (**c**) Curve lines representing virus titres in supernatants. The symbol represents the mean value; the error bars the standard deviation. Micrographs and data are of 3 independent experiments with each condition performed in duplicate cultures. Significant differences between Mock and JEV isolate is indicated by the letters a and b for conditions in the presence of 1:100 and 1:1000 PS, respectively. Statistics are calculated with the Mann-Whitney test (*p < 0.05; **p < 0.01; ***p < 0.001; ****p < 0.0001).

At early periods of exposure e.g. 24 hours, JEV does not alter morphology nor is it cytopathic to human microglia^16^. At later exposure periods of 6 and 8 days, observations under the light microscope demonstrated that both mock- and JEV-treated human microglia were confluent with variable morphologies of flat and elongated or round cells and exerted equivalent granularity (Fig. 1a and b). Therefore, microglia survived long-term exposure to JEV.

Since supernatants of JEV-treated human microglia were free of infectious virus from 6 days of exposure onwards (data not shown), virus recovery was assessed as previously described^16^. To this end, human microglia were pre-treated with JEV for 6, 8, 10 and 12 days and subsequently co-cultured with susceptible target cells such as BHK-21 cells in cell-to-cell contact conditions for 2 additional days in the presence of control porcine serum (Ctrl serum). Virus titres were then determined in supernatants of co-cultures. In the presence of 1:100 of Ctrl serum, virus titres were of 1.41 × 10^4^ TCID_50_/mL (±1.46 × 10^4^), 1.13 × 10^5^ TCID_50_/mL (±2.54 × 10^5^) and 1.82 × 10^1^ TCID_50_/mL (±2.98 × 10^1^) at 6, 8 and 10 days of pre-treatment, respectively. In the presence of 1:1000 of Ctrl serum, virus titres were of 4.62 × 10^2^ TCID_50_/mL (±5.42 × 10^2^), 1.87 × 10^3^ TCID_50_/mL (±2.68 × 10^3^) and 0.61 × 10^1^ TCID_50_/mL (±1.25 × 10^1^) at 6, 8 and 10 days of pre-treatment, respectively. After a pre-treatment of 12 days, no infectious virus was detected with neither 1:100 nor 1:1000 of Ctrl serum. Overall, JEV recovery was statistically significant compared to mock at 6 and 8 days, but not at 10 days after virus pre-treatment. (Fig. 1c).

### Neutralizing anti-JEV antibodies do not completely abrogate cell-to-cell virus transmission and recovery from human microglia

In order to evaluate a possible contact of JEV with the extracellular space during the process of cell-to-cell virus transmission between JEV-infected human microglia and susceptible cells, neutralizing immune serum of pig vaccinated against JEV was employed^22^. Importantly, the potency of antibody-dependent enhancement of infection by JEV^22^ is circumvented by applying antibodies generated in pigs on cells of other species, e.g. human and hamster. Neutralizing titres of immune serum was 1:320 to cell-free JEV^22^ and was used at concentrations of 1:100 and 1:1000. Control (Ctrl) and immune neutralizing (NT) porcine sera were added at time of co-culture of BHK-21 cells and JEV-infected human microglia for 6 or 8 days. Then, levels of intracellular JEV envelop protein (JEV E) were measured in BHK-21 cells and virus titres were determined in supernatants.

**Figure 2:**
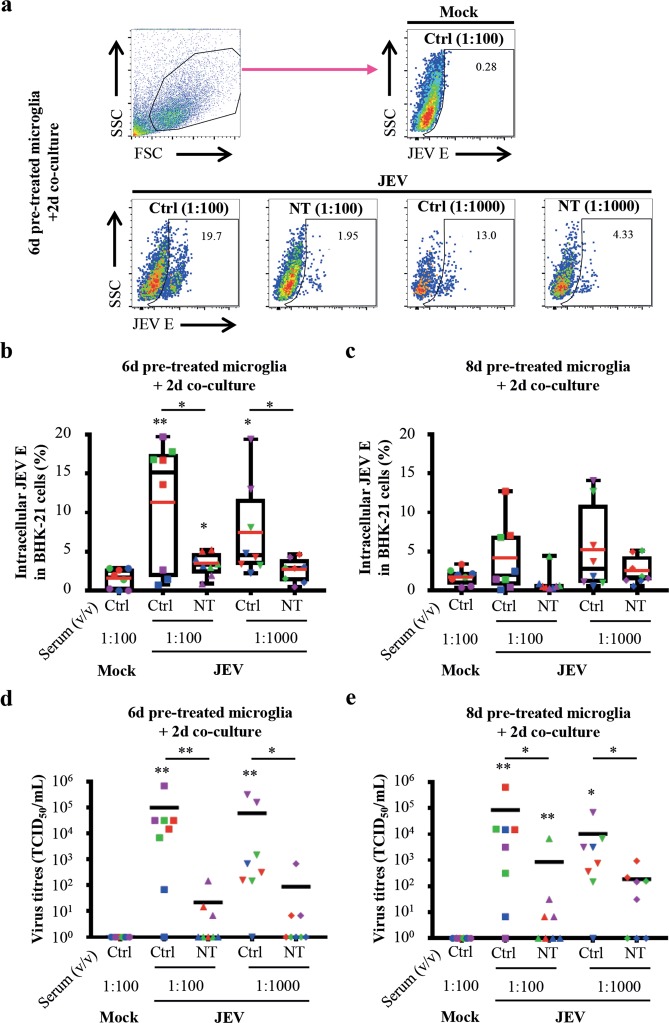
Impact of anti-JEV neutralizing antibodies on virus transmission and recovery. Human microglia were pre-treated with Mock and JEV (Nakayama isolate used at an MOI of 10 TCID_50_/cell) at 37 °C for indicated time-periods. Then, cells were washed with cold PBS and subsequently co-cultured with BHK-21 cells for 2 additional days in the presence of indicated concentrations of control (Ctrl) or neutralizing (NT) porcine serum. (**a**) Upper left panel is a representative density plots showing the gating strategy for the identification of BHK-21 cells based on their FSC/SSC profile. Other panels show representative density plots of intracellular JEV E expression in gated BHK-21cells. Frequency of JEV E^+^-cells is indicated. (**b**,**c**) Box plots representing frequencies of BHK-21 cells expressing JEV E. The symbols represent replicates where each colour is an individual donor. The black line represents the median value, the red line the mean value and the error bars the standard deviation. (**d**,**e**) Scatter dot plots representing virus titres in supernatants. The symbols represent replicates where each colour is an individual donor and the black line represents the mean value. Data are of 4 independent experiments with each condition performed in duplicate cultures. Asterisks on top of a condition show significant differences compared to mock; asterisks on black line show significant differences between the indicated conditions. Statistics are calculated with (**b**,**c**) the t-test or (**d**,**e**) the Mann-Whitney test (*p < 0.05; **p < 0.01; ***p < 0.001; ****p < 0.0001).

In order to evaluate cell-to-cell virus transmission, intracellular JEV E protein was analysed in BHK-21 cells by flow cytometry. BHK-21 cells were gated as big cells based on their forward/scatter (FSC/SSC) profile (Fig. 2a, upper left panel). Intracellular JEV E protein expression was measured on BHK-21 cells, as exemplified from co-cultures of 6d JEV-pre-treated microglia with BHK-21 cells (Fig. 2a). Upon co-culture with 6d JEV-pre-treated human microglia, significant frequencies of JEV E-bearing BHK-21 cells were 11.19% (±8.1) and 7.32% (±6.00) in the presence of 1:100 and 1:1000 Ctrl serum, respectively, as compared to mock. As expected, 1:100 and 1:1000 of NT serum significantly reduced the frequencies of JEV E-bearing BHK-21 cells to 3.35% (±1.48) and 2.63% (±1.49) respectively, as compared to corresponding Ctrl serum conditions. However, the frequencies of JEV E-bearing BHK-21 cells in the presence of 1:100 NT serum remained significant compared to mock (Fig. 2b). Although none of the Ctrl and NT sera conditions showed significance, similar tendencies were pictured upon co-culture of 8d JEV-pre-treated human microglia with BHK-21 cells (Fig. 2c). Overall, neutralizing antibodies did not completely block cell-to-cell virus transmission.

In order to confirm virus recovery, infectious virus particles from culture supernatants were titrated. After 6d cultured JEV-pre-treated microglia, significant virus titres were measured as compared to mock, i.e. 9.97 × 10^4^ TCID_50_/mL (±2.35 × 10^5^) and 5.96 × 10^4^ TCID_50_/mL (±1.17 × 10^5^) in the presence of 1:100 and 1:1000 of Ctrl serum, respectively. As expected, the presence of 1:100 and 1:1000 NT serum decreased virus titres to 2.17 × 10^1^ TCID_50_/mL (±5.09 × 10^1^) and 8.75 × 10^1^ TCID_50_/mL (±2.40 × 10^2^) respectively. This diminution in virus titres was significant compared to corresponding concentration of Ctrl serum (Fig. 2d). Similarly, significant virus titres were measured in 8d cultured JEV-pre-treated microglia compared to mock, i.e. 8.50 × 10^4^ TCID_50_/mL (±2.21 × 10^5^) and 1.03 × 10^4^ TCID_50_/mL (±2.35 × 10^4^) in the presence of 1:100 and 1:1000 of Ctrl serum, respectively. Compared to corresponding concentration of Ctrl serum, virus titres significantly decreased to 8.57 × 10^2^ TCID_50_/mL (±2.41 × 10^3^) and 1.87 × 10^2^ TCID_50_/mL (±3.15 × 10^2^) in the presence of 1:100 and 1:1000 NT serum, respectively. However, only the condition of co-cultures of 8d JEV-pre-treated human microglia and BHK-21 cells in the presence of 1:100 NT serum showed significance compared to mock (Fig. 2e). Therefore, infectious virus could be recovered from co-cultures in the presence of NT serum, demonstrating that *de novo* virus production could overcome neutralizing activities.

### Viral RNA may be a contributing source of infectious viral material upon cell-to-cell virus transmission

**Figure 3:**
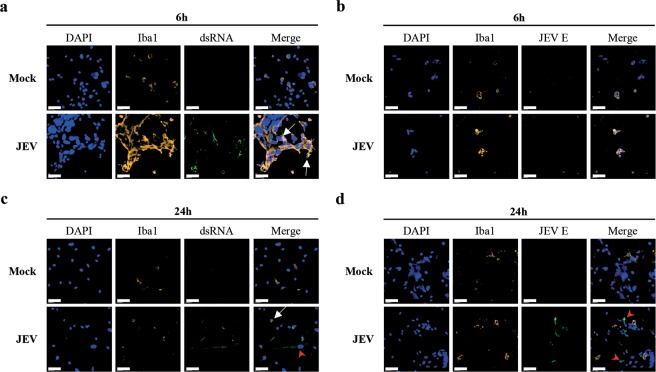
Confocal microscopy analysis of intercellular interactions during virus transmission. Human microglia were adhered with Mock and JEV (Nakayama isolate used at an MOI of 10 TCID_50_/cell) at 37 °C for 2 hours. Cells were then intensively washed with cold PBS and left in culture at 37 °C for 48 hours. Then, cells were intensively washed with PBS before co-culture with BHK-21 cells in the presence of 1:100 Ctrl serum for various time-periods. Cells were stained for the indicated markers and analysed using confocal microscopy. Micrographs showing fluorescence signals upon co-cultures of (**a**,**b**) 6 hours and (**c**,**d**) 24 hours. Scale bars are of 60 μm. White arrows focus on microglia and red arrowheads on BHK-21 cells. Micrographs are representative out of 3 independent experiments.

Considering the fact that human microglia do not produce infectious virus particles, but sustain the replication of viral RNA^16^ and that neutralizing antibodies do not completely block virus transmission and recovery, further experiments aimed to identify the potential source of transmission of infectious viral material upon intercellular interactions. In confocal microscopy experiments of co-cultures, the Iba-1 marker was used to label microglia cells (Iba-1^pos^) and to discriminate from BHK-21 cells (Iba-1^neg^). Immune fluorescence labelling of intracellular dsRNA allowed detection of replicating viral RNA^23,24^ and labelling of intracellular JEV E protein showed viral envelop protein. Mock were free of both dsRNA and JEV E (Fig. 3). Short co-culture periods of 6 hours showed that Iba-1^pos^ microglia contained dsRNA, but no JEV E protein upon pre-treatment with JEV (Fig. 3a and b). After 24 hours of co-cultures, both Iba-1^pos^ microglia and Iba-1^neg^ BHK-21 cells presented dsRNA (Fig. 3c). In parallel, JEV E was mostly found in Iba-1^neg^ BHK-21 cells, but was also observed in Iba-1^pos^ microglia cells (Fig. 3d). This indicates a possible re-infection of the latter cells by *de novo* JEV particles released from the JEV-producing BHK-21 cells.

**Figure 4:**
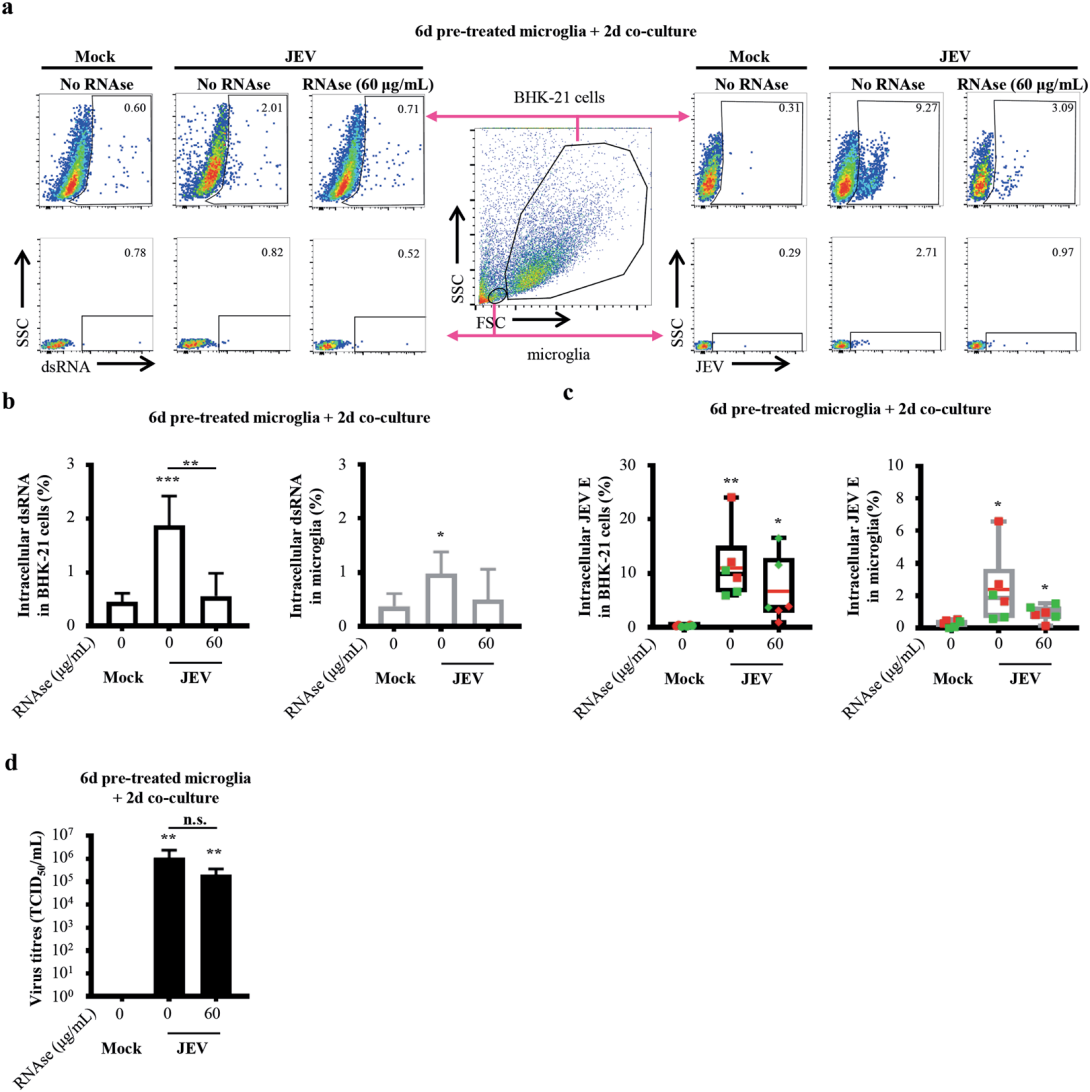
Impact of RNAse A on virus transmission and recovery. Human microglia were pre-treated with Mock and JEV (Nakayama isolate used at an MOI of 10 TCID_50_/cell) at 37 °C for 6 days. Cells were then washed with cold PBS and subsequently co-cultured with BHK-21 cells for 2 additional days in the presence of 1:100 Ctrl serum and indicated concentrations of RNAse A. (**a**) Central panel is a representative density plots of flow cytometry analysis showing the gating strategy for the identification of BHK-21 cells and microglia based on their FSC/SSC profile. Other density plots represents (left panel) intracellular dsRNA and (right panel) JEV E expression in gated (upper panel) BHK-21cells and (lower panel) microglia. Frequency of dsRNA and JEV E^+^ cells is indicated. (**b**) Histogram bars representing frequencies of BHK-21 cells and microglia expressing dsRNA identified as in (**a**). The bar represents the mean value; the error bars the standard deviation. (**c**) Box plots representing frequencies of BHK-21 cells and microglia expressing JEV E identified as in (**a**). The symbols represent replicates where each colour is an individual donor. The black line represents the median value, the red line the mean value and the error bars the standard deviation. (**d**) Histogram bars representing virus titres in supernatants. The bar represents the mean value; the error bars the standard deviation. Data are of 2 independent experiments with each condition performed in duplicate or triplicate cultures. Asterisks on top of a condition show significant differences compared to mock; asterisks on black line show significant differences between the indicated conditions. Statistics are calculated with (**b**,**c**) the t-test or (**d**) the Mann-Whitney test (n.s.: not significant; *p < 0.05; **p < 0.01; ***p < 0.001; ****p < 0.0001).

In order to assess the role of viral RNA in cell-to-cell virus transmission, RNAse A was applied to degrade RNA content in co-cultures such as viral RNA. Using flow cytometry, BHK-21 cells were gated as big cells and microglia as small cells based on their FSC/SSC profile. Then, intracellular dsRNA and JEV E protein were detected in BHK-21 cells and microglia (Fig. 4a). Treatment of co-cultures with RNAse A decreased the frequencies of dsRNA^+^-BHK-21 cells in a dose dependent manner and a dose of 60 μg/mL of RNAse A was used for further experiments (Supplementary Fig. 1a). In the absence of RNAse A, frequencies of dsRNA^+^-BHK-21 cells were of 1.88% (±0.54) whereas 60 μg/mL of RNAse A significantly reduced the frequencies of dsRNA^+^-BHK-21 cells to 0.56% (±0.43). Only conditions in the absence of RNAse A showed significance compared to mock with frequencies of 0.45% (±0.16) (Fig. 4b, left panel). In parallel, the absence of RNAse A revealed frequencies of dsRNA^+^-microglia of 0.98% (±0.39) whereas 60 μg/mL of RNAse A showed frequencies of dsRNA^+^-microglia of 0.50% (±0.56). Again, only conditions in the absence of RNAse A showed significance compared to mock with frequencies of 0.37% (±0.24) (Fig. 4b, right panel).

Deeper analysis of JEV E expression revealed that in the absence of RNAse A, frequencies of JEV E^+^-BHK-21 cells were of 12.39% (±6.93) and 60 μg/mL of RNAse A resulted in frequencies of JEV E^+^-BHK-21 cells of 4.65% (±4.05). Both conditions were significant compared to mock with frequencies of 0.34% (±0.16) (Fig. 4c, left panel). In parallel, the absence of RNAse A showed frequencies of dsRNA^+^-microglia of 2.71% (±2.3) and 60 μg/mL of RNAse A lead to frequencies of dsRNA^+^-microglia of 0.78% (±0.42). Again, both conditions were significant compared to mock with frequencies 0.35% (±0.18) (Fig. 4c, right panel).

However, treatment with RNAse A did not abrogate nor diminished the generation of infectious JEV particles, which titres ranged at 10^5^–10^6^ TCID_50_/mL (Fig. 4d and Supplementary Fig. 1b). Overall, RNAse A effectively reduced intracellular dsRNA in cells but not JEV E. Moreover, the production of *de novo* virus particles overcome RNAse A activity.

### The CX_3_CR1-CX_3_CL1 axis contributes to intercellular interactions for cell-to-cell virus transmission and recovery

**Figure 5:**
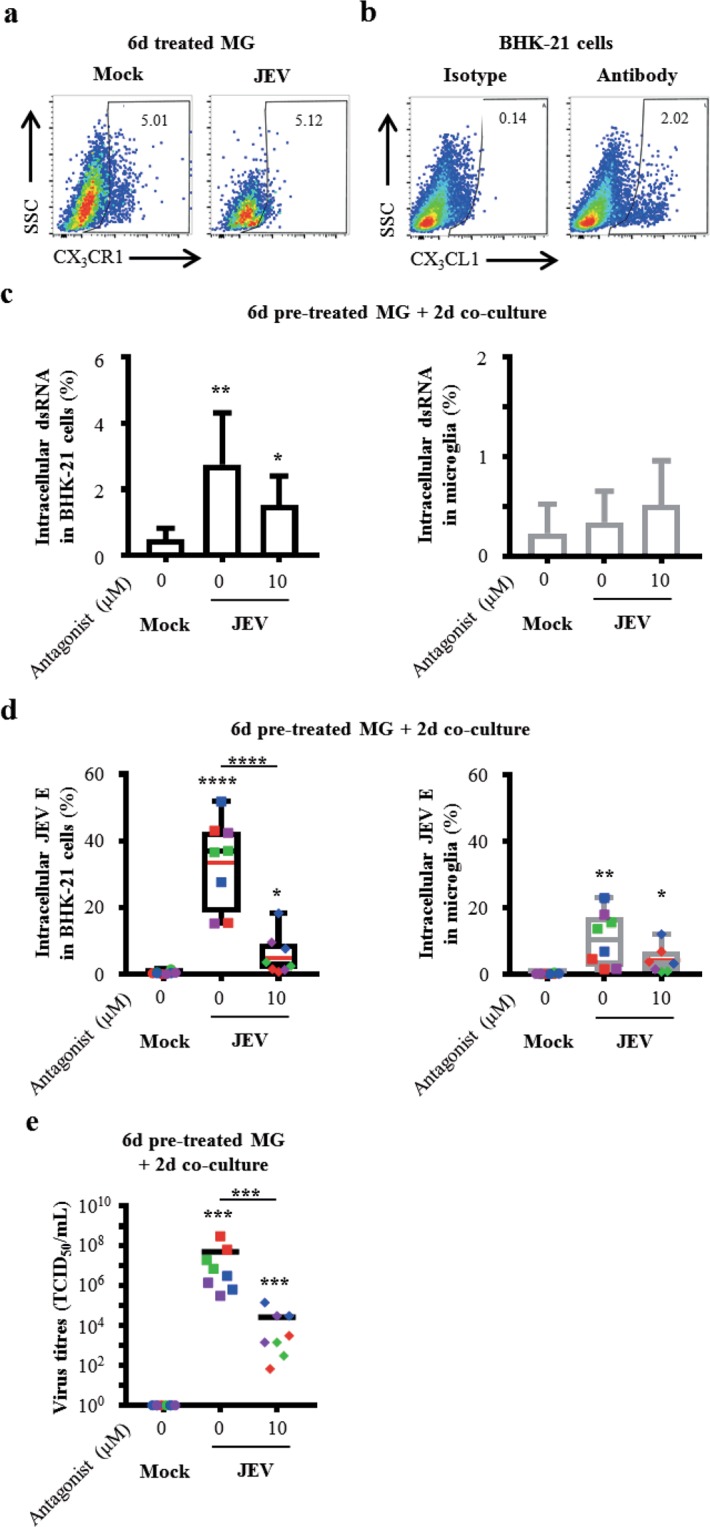
Role of the CX_3_CR1-CX_3_CL1 axis in cell-to-cell virus transmission and recovery. (**a**) Human microglia were treated with Mock and JEV (Nakayama isolate used at an MOI of 10 TCID_50_/cell) at 37 °C for 6 days. Representative density plots of cell surface expression of CX_3_CR1. Frequency of CX_3_CR1^+^ cells is indicated. (**b**) BHK-21 were maintained in culture at 37 °C. Representative density plots of steady state surface expression of CX_3_CL1 by BHK-21 cells. Frequency of CX_3_CL1^+^ cells is indicated. Representative plots are out of 3 independent experiments. (**c**–**e**) Human microglia were pre-treated with Mock and JEV (Nakayama isolate used at an MOI of 10 TCID_50_/cell) at 37 °C for 6 days. Cells were then washed with cold PBS and subsequently co-cultured with BHK-21 cells for 2 additional days in the presence of 1:100 Ctrl serum and indicated concentrations of CX_3_CR1 antagonist. In (**c**,**d**), cells were analysed by flow cytometry for dsRNA and JEV expression in BHK-21 cells and microglia identified as in Fig. 4a. (**c**) Histogram bars representing frequencies of BHK-21 cells and microglia expressing dsRNA. The bar represents the mean value; the error bars the standard deviation. (**d**) Box plots representing frequencies of BHK-21 cells and microglia expressing JEV E. The symbols represent replicates where each colour is an individual donor. The black line represents the median value, the red line the mean value and the error bars the standard deviation. (**e**) Scatter dot plots representing virus titres in supernatants. The symbols represent replicates where each colour is an individual donor and the black line represents the mean value. Data are of 2 or 4 independent experiments with each condition performed in triplicate or duplicate or triplicate cultures. Asterisks on top of a condition show significant differences compared to mock; asterisks on black line show significant differences between the indicated conditions. Statistics are calculated with (**c**,**d**) the t-test or (**e**) the Mann-Whitney test (*p < 0.05; **p < 0.01; ***p < 0.001; ****p < 0.0001).

Upon short period of exposure to JEV (e.g. 2 days), human microglia increase their cell surface expression of CX_3_CR1^16^. After 6 days, ~5% of human microglia expressed CX_3_CR1 on their cell surface upon JEV exposure and was comparable to mock (Fig. 5a). In counterpart, ~2% of BHK-21 cells expressed CX_3_CL1 on their surface, the ligand for CX_3_CR1 (Fig. 5b). Therefore, the role of the CX_3_CR1-CX_3_CL1 axis upon intercellular interactions during virus transmission was investigated using a non-competitive, potent and highly specific antagonist for CX_3_CR1 in co-cultures^25,26^. Importantly, doses of 10 μM of CX_3_CR1 antagonist did not affect the capacity of virus propagation by BHK-21 cells (Supplementary Fig. 2a). Overall, treatment with CX_3_CR1 antagonist decreased the frequencies of JEV E-expressing cells and virus titres in a dose-dependent manner. Since 10 μM of CX_3_CR1 antagonist efficiently inhibited virus transmission and recovery, this dose was employed for further analysis (Supplementary Fig. 2b and c).

Cell-to-cell virus transmission was investigated by the detection of intracellular dsRNA and JEV E protein in BHK-21 cells and microglia by flow cytometry after 2 days of co-cultures since earlier time-periods of co-cultures did not allow quantification of dsRNA and JEV E protein (Supplementary Fig. 3a and b). In the absence of CX_3_CR1 antagonist, frequencies of dsRNA^+^-BHK-21 cells were of 2.75% (±1.57) and treatment with 10 μM of CX_3_CR1 antagonist lead to reduced frequencies of dsRNA^+^-BHK-21 cells to 1.54% (±0.87). Both conditions showed significance compared to mock with frequencies of 0.49% (±0.34) (Fig. 5c, left panel). In parallel, frequencies of dsRNA^+^-microglia were of 0.35% (±0.31) and 0.51% (±0.45) in the absence and the presence of 10 μM of CX_3_CR1 antagonist, respectively. No significance was found compared to mock with frequencies of 0.23% (±0.29) (Fig. 5c, right panel).

Analysis of JEV E expression showed frequencies of JEV E-expressing BHK-21 cells of 33.64% (±13.23) in the absence of CX_3_CR1 antagonist. As expected, treatment with 10 μM of CX_3_CR1 antagonist significantly reduced the frequencies of JEV E-expressing BHK-21 cells to 5.63% (±6.03). Both conditions were significant compared to mock with frequencies of 0.42% (±0.47) (Fig. 5d, left panel). In parallel, frequencies of JEV E^+^-microglia were of 10.62% (±8.07) and 4.17% (±4.09) in the absence and the presence of 10 μM of CX_3_CR1 antagonist, respectively. Both conditions were significant in comparison to mock showing frequencies of 0.17% (±0.19) (Fig. 5d, right panel).

In addition, recovery of infectious virus particles was evaluated by virus titration in supernatants. Importantly, recovery of infectious virus particles was confirmed in the absence of CX_3_CR1 antagonist by significant titres of 5.21 × 10^7^ TCID_50_/mL (±1.09 × 10^8^) when compared to mock. In line with previous observations by flow cytometry, treatment with 10 μM of CX_3_CR1 antagonist significantly reduced virus titres to 2.71 × 10^4^ TCID_50_/mL (±5.04 × 10^4^) but these titres remained significant compared to mock (Fig. 5e). Upon co-culture of 8d JEV-pre-treated human microglia with BHK-21 cells, similar picture were observed although no significant changes were obtained regarding the frequencies of JEV E-expressing cells from the various conditions. Otherwise, virus titres in the absence of the CX_3_CR1 antagonist were significant in comparison to mock. Importantly, treatment with 10 μM of CX_3_CR1 antagonist significantly reduced the content of infectious virus particles in BHK-21 cells (Supplementary Fig. 4a and b). Overall, antagonistic treatment of CX_3_CR1 inhibited cell-to-cell virus transmission and recovery.

### The CX_3_CR1-CX_3_CL1 axis may influence the type of intercellular interactions

**Figure 6:**
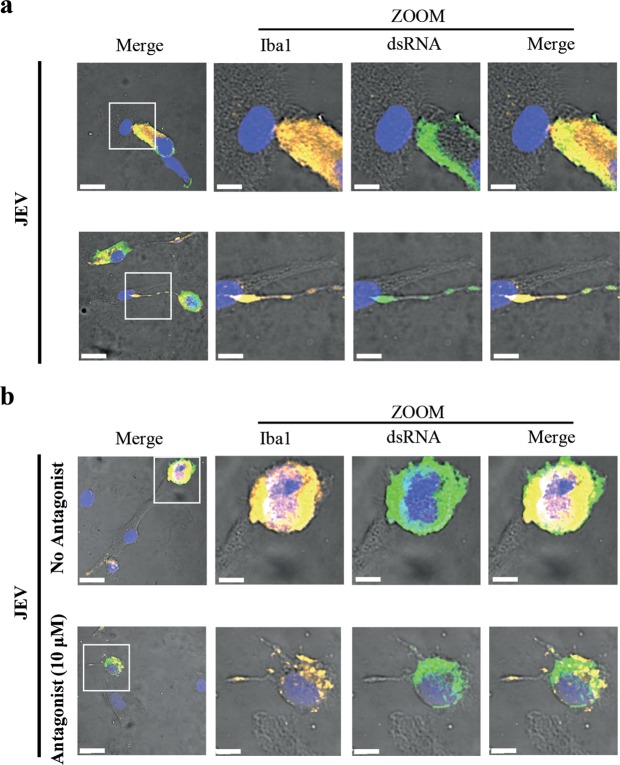
Role of the CX_3_CR1-CX_3_CL1 axis on intercellular interactions. Human microglia were adhered with JEV (Nakayama isolate used at an MOI of 10 TCID_50_/cell) at 37 °C for 2 hours. Cells were then intensively washed with cold PBS and left in culture at 37 °C for 48 hours. Cells were intensively washed with PBS and co-cultured with BHK-21 cells. In (**a**), co-cultures were performed in the presence of 1:100 Ctrl serum for 6 hours. In (**b**), co-cultures were performed in the presence of 1:100 Ctrl serum and in the absence or the presence of CX_3_CR1 antagonist for 6 hours. Cells were stained for the indicated markers and analysed using confocal microscopy. (**a**,**b**) Micrographs showing fluorescence signals on bright field overlay focusing on intercellular interactions. Zoom of white square in left panel is presented. Scale bars are of 20 μm in left panels and of 8 μm in zoom panels. Micrographs are representative out of 3 independent experiments.

JEV transmission and recovery requires cell-cell contact^16^. Already at short co-culture periods, two types of cell-to-cell interactions were observed between Iba-1^pos^ microglia-containing dsRNA and Iba-1^neg^ BHK-21 cells. In the majority of the observations, the two cell types were close to each other with a large portion of the surface cell membranes in contact (Fig. 6a, upper panel), indicating virological synapses^27^. To a lesser extent, the two cell types were more distant from each other, but forming thin membrane protrusions^27^ containing dsRNA in the Iba-1^pos^ microglia tubular extensions, which interacted with BHK-21 cells (Fig. 6a, lower panel).

Co-cultures in the presence or the absence of the CX_3_CR1 antagonist were also analysed by confocal microscopy using a similar approach as described before. In both conditions, Iba-1^pos^ microglial cells contained dsRNA and interacted with Iba-1^neg^ BHK-21 cells. Intercellular interactions were abundant and of a virological synapses appearance^27^ in the absence of the antagonist. Although the frequency had substantially decreased, membrane protrusions^27^ were occasionally observed in the presence of CX_3_CR1 antagonist (Fig. 6b).

## Discussion

Microglia are the first line of defence against CNS insults^14^. In the context of JE, microglial cells are the major producers of antiviral inflammatory mediators within the CNS^28^. Indeed, human microglia produce various inflammatory mediators upon exposure to JEV^16^. However, microglia may also serve as reservoir for JEV, since they sustain viral replication for long period after JEV exposure^16,17,29^. For cell-to-cell JEV transmission, the present study highlights the potential involvement of human microglia in JEV propagation and spreading in the brain. Cell-to-cell contact-dependent virus transmission may substantially contribute to the spread of the virus infection^30^. Moreover, virus recovery after late periods may contribute to persistent JEV infection. Persistency of JEV in the human CNS has been suggested, since infectious virus can be found in the cerebrospinal fluid of patients beyond the third week of illness^31^. Although it is unknown whether JEV can be reactivated, a biphasic pattern of JE has been described with a relapse of the disease 14–32 days after illness recovery^32^. Thus, JEV transmission by microglia may initiate another cycle of JEV activation leading to JE relapse in persistent infection.

Cell-to-cell contact-dependent virus transmission is a mechanism used for viral escape to immunity. In our study, antibodies efficiently neutralized cell-free JEV^22^, but were inefficient in blocking cell-to-cell transmission of microglia-associated JEV. First, resistance to neutralizing antibodies may result from the inefficacy of a certain subset of neutralizing antibodies to inhibit cell-to-cell virus transmission^27^. Otherwise, resistance to neutralizing antibodies may be due to the inaccessibility of antibodies within intercellular interaction structures. Cytosol-to-cytosol connectivity between cells, as well as cytoplasmic connections-containing vesicles prevent exposure of viral material to the extracellular space^33^. Finally, the present study used antibodies derived from pigs treated with a lentivirus-based vaccine against JEV G3 prM and E proteins^34^. Therefore, other potential sources of viral material such as genomic material and capsid protein are not recognized by antibodies-derived vaccine.

The present study suggests that genomic viral RNA is contributing, but not the exclusive source of infectious viral material, to cell-to-cell virus transmission. In various studies, virus particles have been the source of infectivity in cell-to-cell virus transmission^35–37^. Here, the absence of JEV E protein in JEV-infected microglia cells reflects the inability of forming virus particles. Thus the absence of virus particles, but the detection of intracellular dsRNA demonstrates a replicative form of JEV viral RNA, similar to other ssRNA + viruses^24^. Viral RNA in its dsRNA and/or ssRNA forms may be sufficient for cell-to-cell transmission and the recovery of infectious virus including JEV^38,39^. Despite decreasing intracellular dsRNA content, treatment with RNAse A does not abrogate infectious JEV recovery. Because RNAse A degrades ssRNA, this suggests that dsRNA is more likely to be the source of virus material than ssRNA. However, other viral factors may also contribute to cell-to-cell virus transmission.

A cell-to-cell contact-dependent mode of transmission of viral genomic RNA represents an alternative mechanism enabling the generation of *de novo* viable virus particles^33^. Human microglia are inefficient to generate infectious virus particles and recovery of microglia-associated JEV by target cells depends on cell contact^16^. JEV induces virological synapses in a contact-dependent mode of virus transmission between virus-pulsed dendritic cells and T cells^35^. Here, microglia may use virological synapses and membrane protrusions such as nanotubes and/or filopodia structures for intercellular interactions. Virological synapses require the contribution of adhesion molecules together with microtubules and actin cytoskeleton for stabilization, whereas membrane extension are actin-rich structures^27^.

Importantly, virus transmission from JEV-treated microglia to target cells involves CX_3_CR1-CX_3_CL1 interaction. In the CNS, microglia express CX_3_CR1 whereas neurons express CX_3_CL1 and the CX_3_CR1-CX_3_CL1 axis is a main regulator of chemotaxis and cross-communication between microglia and neurons^18^. In the present study, low levels of CX_3_CR1 expression on microglia allowed cell-to-cell virus transmission indicating an extreme sensitivity of this mechanism. The fact that JEV-treated microglia transiently up-regulates surface expression of CX_3_CR1^16^ suggests that cell contact-mediated virus transmission may significantly contribute to infection of neuronal cells at early time of infection. Therefore, the CX_3_CR1-CX_3_CL1 axis could be a potential therapeutic target candidate in JEV-infected patients. However, CX_3_CL1 is crucial in the inhibition of the production and neurotoxicity of JEV-infected microglia-derived inflammatory factors^40,41^. Moreover, polymorphism of CX_3_CR1^42^ may influence the specificity of potential therapeutics. Polymorphism of CX_3_CR1 may also explain the differences observed in virus transmission and recovery between cells of blood donors. Nevertheless, the CX_3_CR1 antagonist did not completely abrogate cell-to-cell JEV transmission indicating that additional or alternative factors may be involved in transmission. For example, DC-SIGN, which is expressed in human microglia^43,44^, promotes dendritic cell-to-T cell JEV transmission^35^ and mediates cellular modifications such as cytoskeleton remodelling promoting filopodia extension^45^.

## Methods

### Authorization, ethics approval and consent to participate

The Federal Office for the Environment (FOEN, Bern, Switzerland) provided authorization for the collection of human samples and manipulation of the various cells and viruses (authorization number A130522). Ethics approval 034/13-CER-FR for the characterization and differentiation of human white blood cells has been granted by the Ethics Committee of the Canton of Fribourg according to corresponding laws and regulations, based on the Declaration of Helsinki. No consent form was required as the buffy coats were provided from the Swiss Red Cross Blood Bank. All samples were analysed anonymously.

### Antibodies and stains

The viral envelop protein was detected using the pan-immune anti-flavivirus antibody (mouse IgG1/IgG2a, clone ATCC-HB-112 D1-4G2-4-15 hybridoma, ATCC, Wesel, Germany) and double stranded RNA (dsRNA) was detected with the anti-dsRNA J2 antibody (mouse IgG2a, Scicons, Budapest, Hungary). Cell markers were detected using anti-human antibodies against CX_3_CL1 (rabbit polyclonal IgG, clone PA5-23062, Thermofischer Scientific, Waltham, MA), Iba-1 (rabbit polyclonal IgG, Wako, Richmond, VA) and fluorescent-labelled CX_3_CR1-R-Phycoerythrin (rat IgG2b, clone 2A9-1, Miltenyi Biotec GmbH, Bergisch-Gladbach, Germany).

The following secondary antibodies were used for staining: donkey anti-rabbit IgG fluorescent-labelled Alexa Fluor 647 (AF647) (Abcam, Cambridge, UK) and goat anti-mouse IgG2a fluorescent-labelled AF647 for flow cytometry, as well as goat anti-mouse IgG2a fluorescent-labelled AF488 and donkey anti-rabbit IgG fluorescent-labelled AF546 for confocal microscopy (all obtained from Thermofischer Scientific if not mentioned). Nuclei were stained using DAPI (Sigma-Aldrich, Saint Louis, MO). All concentrations for the use of the antibodies and stains were optimized in our laboratory.

### Cell line culture

Baby Hamster Kidney-21 cells (BHK-21 cells, fibroblasts) ([C-13], ATCC) were cultured in Glasgow’s Minimum Essential Medium (GMEM) (Thermofischer Scientific) supplemented with 10% (v/v) Fetal Bovine Serum (FBS) (Biowest, Nuaillé, France) and Tryptose Phosphate Broth solution (Sigma-Aldrich) at 37 °C and 5% CO_2_.

### Virus preparation and titration

JEV Nakayama isolate (National collection of pathogenic viruses, NCPV, Salibury, UK) was propagated in BHK-21 cells as previously described^16^. Briefly, 80% confluent BHK-21 monolayer cell culture was infected with JEV suspended in RPMI-1640 GlutaMAX^TM^-I medium (Thermofischer Scientific) supplemented with 2% FBS and cultured until cytopathogenic effects (approx. 36–48 hours). Virus stock suspension was obtained after disruption of remaining cells by freezing and centrifugation of cell soup at 3000 g at 4 °C for 30 minutes to eliminate cell debris. In parallel, mock was prepared accordingly from uninfected BHK-21 cells and served as negative control of virus exposure in experiments.

Virus titres of virus stocks and experimental supernatants were determined by end-point titration. Briefly, 10-fold serial dilutions of suspension in GMEM supplemented with 10% FBS and Tryptose Phosphate Broth solution were applied on BHK-21 cells at 37 °C, 5% CO_2_. After 36–48 hours, intracellular viral particles were detected with the pan-immune anti-flavivirus antibody followed by peroxidase enzymatic reaction.

### Generation of human microglial cells

Human blood monocyte-derived microglia were generated from buffy coats of anonymous healthy donors obtained from Blutspendedienst (Bern, Switzerland) using a protocol adapted from^46^. Human peripheral blood mononuclear cells (PBMC) were isolated from buffy coat after Ficoll-Paque density gradient centrifugation (1.077 g/L, Amersham Pharmacia Biotech AG, Dubendorf, Switzerland). Monocytes were enriched using positive selection of CD14^+^ cells with selection columns and magnetic sorting system (Miltenyi Biotech GmbH). CD14^+^ monocytes were cultured at a concentration of 0.5 × 10^6^ cells/mL in RPMI-1640 GlutaMAX^TM^-I medium. Medium was supplemented with antibiotic/antimycotic and bioactive human recombinant granulocyte macrophage colony-stimulating factor (GM-CSF) (10 ng/mL), macrophage colony-stimulating factor (M-CSF) (10 ng/mL), nerve growth factor (NGF)-β (10 ng/mL) and CC chemokine ligand 2 (CCL2) (50 ng/mL) (all purchased from Miltenyi Biotech GmbH), at 37 °C and 5% CO_2_ for 7 days. Half of the medium was renewed after the 3 days of culture.

### Treatment of human microglial cells with JEV and co-culture with susceptible target cells

Human microglia were treated with Mock or JEV (at a multiplicity of infection (MOI) of 10 TCID_50_/cell) in RPMI-1640 GlutaMAX^TM^-I medium at 37 °C and 5% CO_2_ during various time-periods. Supernatants were collected and pre-treated human microglia were washed 5 times with cold PBS. Both supernatants and last washing was verified negative for infectious JEV by end-point titration. Subsequently, susceptible target BHK-21 cells were co-cultured with pre-treated human microglia in RPMI-40 GlutaMAX^TM^-I medium, at 37 °C in 5% CO_2_. Co-cultures were performed in commercial porcine serum (Thermofischer Scientific). After various time-periods of co-culture, supernatants and cells were collected for further analysis.

In some experiments, immune porcine serum of vaccinated pigs with neutralizing activity against Nakayama isolate at a titre of 1:320^22^ and previously described commercial porcine serum was used as control. In other experiments, CX_3_CR1 antagonist (AZD8797, Axon Biochemicals, Groningen, The Netherlands) suspended in DMSO (Thermofischer Scientific) and/or RNAse A (Macherey Nagel, Düren, Germany) suspended in PBS were used. DMSO and PBS were respectively used as controls. These reactive were put on top of treated microglial cells before the addition of BHK-21 cells for co-cultures.

### Flow cytometry

Cells were analysed using multi-colour flow cytometry FACSCanto II instrument (BD Biosciences, San Jose, CA). Data were analysed using FlowJo Software (Data analysis Software, Ashland, OR).

### Bright field and confocal microscopy

For conventional bright field microscopy, cells were cultured in flat bottom well plates. Cells were analysed and photographed using an EVOS XL Core digital inverted microscope with cell imaging system (Thermofischer Scientific). The image acquisitions were performed with the 10x and 40x objectives.

For confocal microscopy, cells were alternatively cultured in labteck or on autoclaved coverslips. After staining, slides were then mounted using Mowiol and left overnight at 4 °C for solidification. Cells were analysed and photographed using a confocal microscope A1 combined with an ECLIPSE Ti inverted microscope and a digital imaging NIS-Elements AR 3.30.02software (Nikon AG, Egg, Switzerland). The image acquisitions were performed with the 20x and 40x objectives. Images were treated with Imaris 9.1 software (Bitplane AG, Zürich, Switzerland), applying background subtraction, threshold applications, gamma correction and maxima.

### Biosafety

All experiments using JEV were conducted in a biosafety level 3 bio-containment. After inactivation with 3% paraformaldehyde solution at room temperature for 15 minutes, samples were further analysed by flow cytometry and microscopy.

### Statistical analysis

Significant differences were determined with GraphPad Prism 6 software (GraphPad software Inc., La Jolla, CA) using the student t-Test (p < 0.05) or the Mann-Whitney Test (p < 0.05).

## Supplementary information

Supplementary Figures

## Data Availability

The datasets are available upon request
