# Supplementary material for: CX3CR1-CX3CL1-dependent cell-to-cell Japanese encephalitis virus transmission by human microglial cells
Source: Sci Rep. 2019 Mar 18;9:4833. doi: 10.1038/s41598-019-41302-1 (PMC6423114; doi:10.1038/s41598-019-41302-1)
Supplement: Supplementary file 1 — Supplementary Figures [file 41598_2019_41302_MOESM1_ESM.pdf]

**$CX_3CR1$-$CX_3CL1$-dependent cell-to-cell Japanese encephalitis virus transmission by human microglial cells**

Nils Lannes[1*], Obdullio Garcia-Nicolas[2,3], Thomas Démoulins[2,3], Artur Summerfield[2,3] and Luis Filgueira[1]

[1] Unit of Anatomy, Department of Medicine, University of Fribourg, Route Albert-Gockel 1, Fribourg, Switzerland
[2] Institute of Virology and Immunology, Sensemattstrasse 293, Mittelhäusern, Switzerland
[3] Department of Infectious Diseases and Pathobiology, Vetsuisse Faculty, University of Bern, Langassstrasse 122, Bern, Switzerland

Authors' e-mail: NL: nils.lannes@unifr.ch; OGN: obdullio.garcia-nicolas@ivi.admin.ch; TD: thomas.demoulins@ivi.admin.ch; AS: artur.summerfield@ivi.admin.ch; LF: luis.filgueira@unifr.ch

Corresponding author: Nils Lannes; e-mail: nils.lannes@unifr.ch

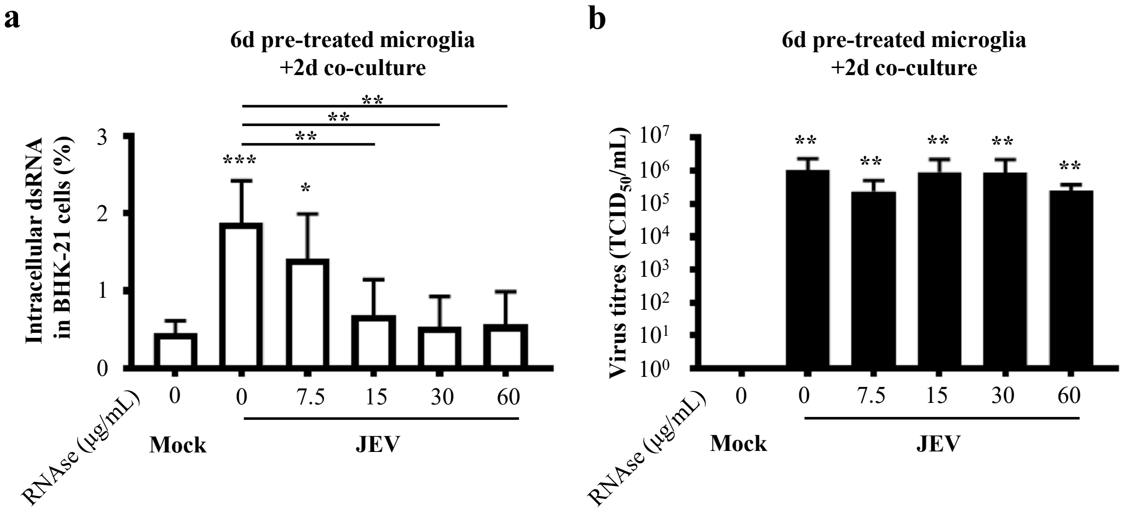


**Supplementary Figure 1. Dose dependent impact of RNAse A on virus transmission and recovery.** Human microglia were pre-treated with Mock and JEV (Nakayama isolate used at an MOI of 10 TCID50/cell) at 37°C for 6 days. Cells were then washed with cold PBS and subsequently co-cultured with BHK-21 cells for 2 additional days in the presence of 1:100 Ctrl serum and indicated concentrations of RNAse A. **(a)** Histogram bars representing frequencies of BHK-21 cells expressing dsRNA identified as in Figure 4a upon flow cytometry analysis. The bar represents the mean value; the error bars the standard deviation. **(b)** Histogram bars representing virus titres in supernatants. The bar represents the mean value; the error bars the standard deviation. Data are of 2 independent experiments with each condition performed in duplicate or triplicate cultures. Asterisks on top of a condition show significant differences compared to mock; asterisks on black line show significant differences between the indicated conditions. Statistics are calculated with **(a)** the t-test or **(b)** the Mann-Whitney test (* : $p<0.05$; ** : $p<0.01$; *** : $p<0.001$; **** : $p<0.0001$).

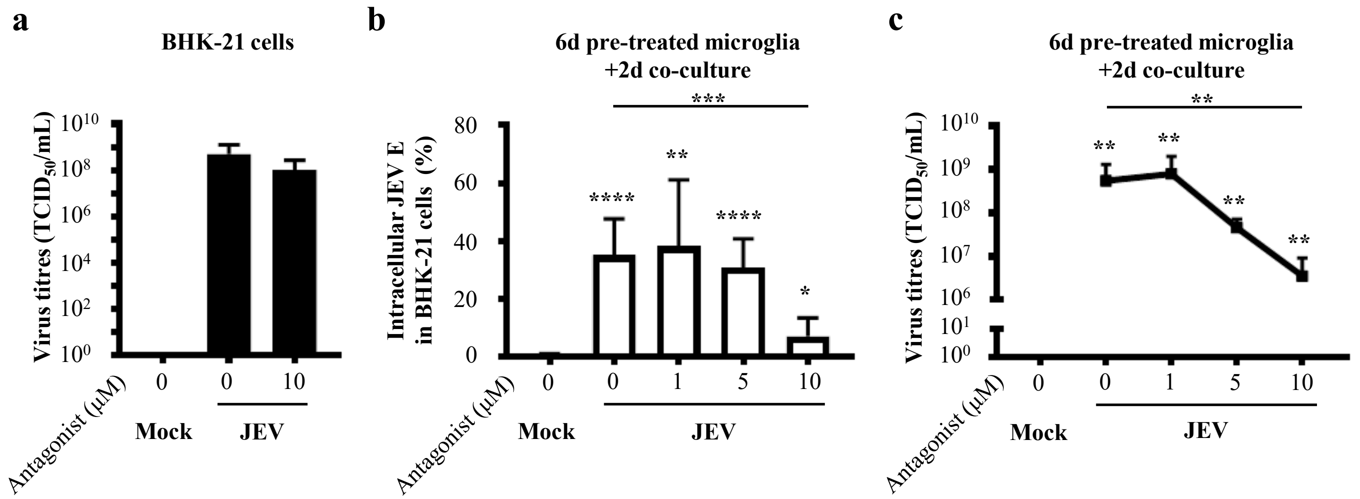


**Supplementary Figure 2. Dose dependent impact of $CX_3CR1$ antagonist on viral propagation in BHK-21, virus transmission and recovery. (a)** BHK-21 were treated with Mock and JEV (Nakayama isolate used at an MOI of 0.01 $TCID_{50}$/cell) at 37°C for 2 days. Histogram bars representing virus titres in supernatants. The bar represents the mean value; the error bars the standard deviation. Data are of 3 independent experiments. **(b, c)** Human microglia were pre-treated with Mock and JEV (Nakayama isolate used at an MOI of 10 $TCID_{50}$/cell) at 37°C for 6 days. Cells were then washed with cold PBS and subsequently co-cultured with BHK-21 cells for 2 additional days in the presence of 1:100 Ctrl serum and indicated concentrations of $CX_3CR1$ antagonist. **(b)** Histogram bars representing frequencies of BHK-21 cells expressing JEV E identified as in Figure 4a upon flow cytometry analysis. The bar represents the mean value; the error bars the standard deviation. **(c)** Curve lines representing virus titres in supernatants. The symbol represents the mean value; the error bars the standard deviation. Data are of 3 independent experiments with each condition performed in duplicate or triplicate cultures. Asterisks on top of a condition show significant differences compared to mock; asterisks on black line show significant differences between the indicated conditions. Statistics are calculated with **(b)** the t-test or **(a, c)** the Mann-Whitney test (* : $p<0.05$; ** : $p<0.01$; *** : $p<0.001$; **** : $p<0.0001$).

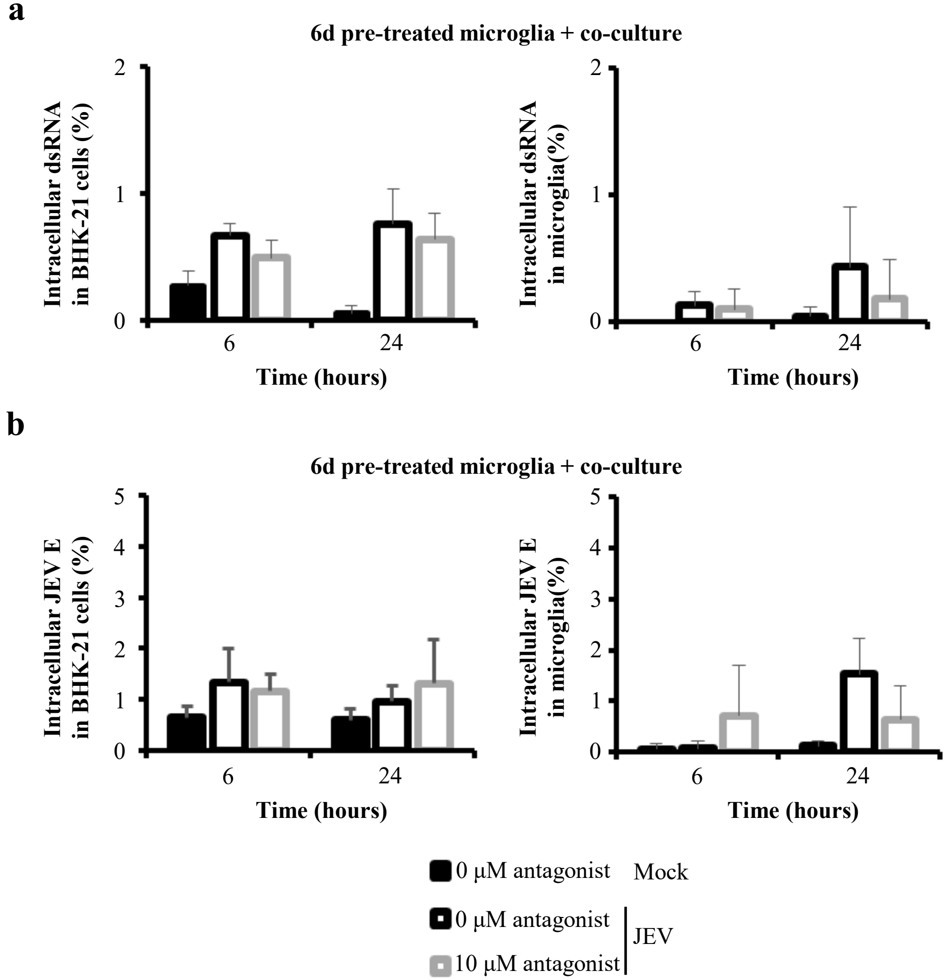


**Supplementary Figure 3. Impact of $CX_3CR1$ antagonist on dsRNA and JEV E expression in BHK-21 cells and microglia at early time periods of incubation.** Human microglia were pre-treated with Mock and JEV (Nakayama isolate used at an MOI of 10 $TCID_{50}$/cell) at 37°C for 6 days. Cells were then washed with cold PBS and subsequently co-cultured with BHK-21 cells for indicated time period in the presence of 1:100 Ctrl serum and indicated concentrations of $CX_3CR1$ antagonist. Cells were analysed by flow cytometry. Histogram bars representing frequencies of BHK-21 cells and microglia expressing **(a)** dsRNA and **(b)** JEV E identified as in Figure 4a. Data represent one experiments done in triplicate of cultures.

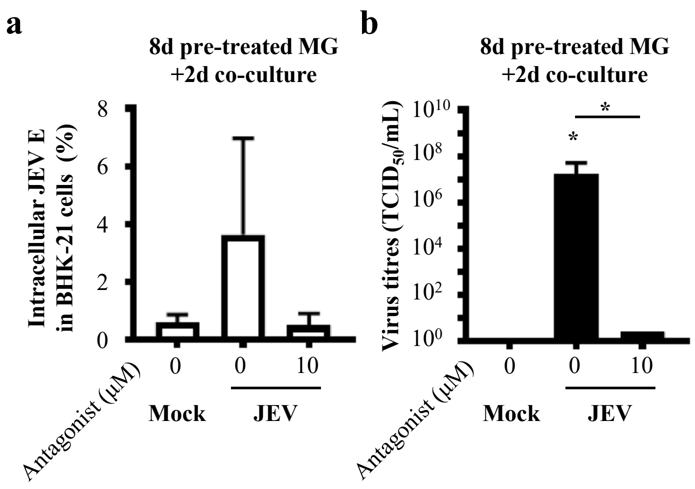


**Supplementary Figure 4. Impact of $CX_3CR1$ antagonist on virus transmission and recovery by 8d pre-treated human microglia.** Human microglia were pre-treated with Mock and JEV (Nakayama isolate used at an MOI of 10 $TCID_{50}$/cell) at 37°C for 8 days. Cells were then washed with cold PBS and subsequently co-cultured with BHK-21 cells for 2 additional days in the presence of 1:100 Ctrl serum and indicated concentrations of $CX_3CR1$ antagonist. **(a)** Histogram bars representing frequencies of BHK-21 cells expressing JEV E identified as in Figure 4a upon flow cytometry analysis. **(b)** Histogram bars representing virus titres in supernatants. The bar represents the mean value; the error bars the standard deviation. Data are of 2 independent experiments with each condition performed in duplicate or triplicate cultures. Asterisks on top of plot show significant differences compared to mock; asterisks on black line show significant differences between the indicated conditions. Statistics are calculated with **(a)** the t-test or **(b)** the Mann-Whitney test (* : $p<0.05$; ** : $p<0.01$; *** : $p<0.001$; **** : $p<0.0001$).
